# Complex renal cysts (Bosniak ≥IIF): interobserver agreement, progression and malignancy rates

**DOI:** 10.1007/s00330-020-07186-w

**Published:** 2020-08-27

**Authors:** James Lucocq, Sanjay Pillai, Richard Oparka, Ghulam Nabi

**Affiliations:** 1 Department of Urology, Research Division of Imaging Sciences and Technology, School of Medicine, Ninewells Hospital and Medical School, Dundee, Scotland UK; 2 Department of Radiology, Research Division of Imaging Sciences and Technology, School of Medicine, Ninewells Hospital and Medical School, Dundee, Scotland UK; 3 Department of Pathology, Research Division of Imaging Sciences and Technology, School of Medicine, Ninewells Hospital and Medical School, Dundee, Scotland UK

**Keywords:** Neoplasms, Kidney, Cysts

## Abstract

**Objective:**

The objective was to assess the interobserver agreement rate, progression rates and malignancy rates in the assessment of complex renal cysts (≥ Bosniak IIF) using a population-based database.

**Methods:**

A regional database identified 452 complex renal cysts in 415 patients between 2009 and 2019. Each patient was tracked and followed up using a unique identifier and deterministic linkage methodology. The interobserver agreement rate between radiologists was calculated using a weighted kappa statistic. Progression and malignancy rates of cysts (Bosniak ≥IIF) over the 11-year period were calculated.

**Results:**

The linear-weighted kappa value was 0.69 for all complex cysts. The rate of progression and regression of Bosniak IIF cysts was 4.6% (7/151) and 3.3% (5/151), respectively. All malignant IIF cysts progressed within 16 months of diagnosis. The malignancy rate of surgically resected Bosniak III and IV cysts was 79.3% (23/29) and 84.5% (39/46), respectively. Of all malignant tumours, 73.8% and 93.7% were of low ISUP grade and low stage, respectively.

**Conclusions:**

This study further confirms that there is a good degree of agreement between radiologists in classifying complex renal masses using the Bosniak classification. The progression rate of Bosniak IIF cysts is low, but the malignancy rates of surgically resected Bosniak IIF, III and IV cysts are high. Benign cysts are frequently resected, and a very high proportion of histopathologically confirmed cancers in complex renal cysts are of low grade and stage.

**Key Points:**

***•*** *There is a good degree of agreement between radiologists in classifying complex renal masses using the Bosniak classification.*

***•*** *The rate of progression of Bosniak IIF cysts is low, and malignant cysts progress early during surveillance. Although the malignancy rates of resected Bosniak IIF, III and IV cysts are high, the rate of benign cyst resection is significant.*

**Electronic supplementary material:**

The online version of this article (10.1007/s00330-020-07186-w) contains supplementary material, which is available to authorized users.

## Introduction

The Bosniak classification stratifies renal cysts using radiological appearances to determine malignancy risk. Radiological features indicative of malignancy include irregular septa, nodular changes, wall thickening and significant enhancement. Categorising renal cysts accurately using the Bosniak classification can be challenging due to its subjective criteria and variations in observer experience [1, 2]. Within the literature, there remains a large degree of variation in the reported interobserver agreement rate between radiologists. Consequently, the risk of cyst misclassification and inappropriate surgical or conservative management is unclear and warrants further investigation [3, 4].

The management of Bosniak cysts is dependent on cyst category. Grounded by malignancy rates, the literature supports discharge of Bosniak I and II cysts, imaging follow-up of Bosniak IIF cysts and surgical intervention for Bosniak III and IV cysts. Interval imaging follow-up of Bosniak IIF cysts is necessary because of the risk of cyst progression and malignancy [5–7]. Although the rate of progression is variable, a high malignancy rate of progressed Bosniak IIF cysts has been reported necessitating follow-up [5, 8]. The likelihood and time to progression are undetermined in the literature, but a minimum of 1 year for uncomplicated and up to 4 years for more complex Bosniak IIF cysts has been suggested [1, 9]. Further studies are required to establish the rate of progression, rate of malignancy and appropriate length of follow-up.

The malignancy risk of resected Bosniak III cysts is highly variable in the literature and is reported to be between 33 and 84%; thus, benign cysts are frequently resected unnecessarily [8, 10–12]. When classified as malignant by histopathology, Bosniak III cysts are frequently low ISUP grade and stage [7, 13, 14]. Although resection of Bosniak III cysts is advised for most patients, additional research, in particular with longer follow-up, is required to establish the rate of benign cyst resection and the frequency of low-grade/stage tumours in order to improve surgical decision-making.

The objectives of this study were:

- To assess the interobserver agreement rate between radiologists to evaluate the reliability of the Bosniak classification system and the risk of inappropriate management.
- To evaluate the progression rate of Bosniak IIF cysts and establish an appropriate length for follow-up of Bosniak IIF cysts.
- To determine the malignancy rate of resected complex cysts (Bosniak ≥IIF), particularly Bosniak III cysts.

## Methods

### Definition of population cohort

**Fig. 1:**
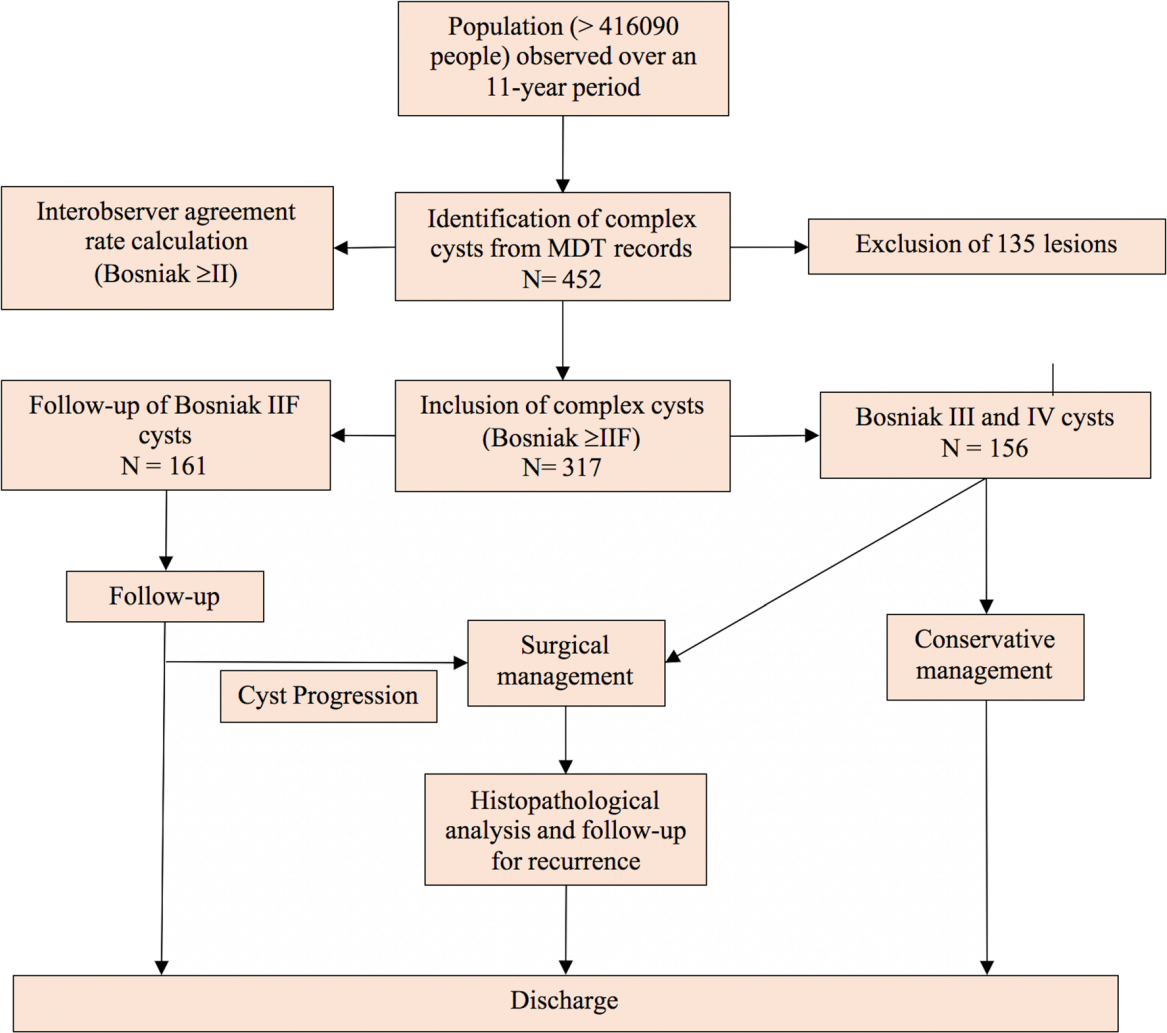
Study design

A regional database stores the health records of patients with complex renal cysts diagnosed in a defined geographical area with a population of more than 416,090 people, based on mid-year 2017 population estimates [15]. The stable population with less than 1% migration rate within the area is registered with the health board through a unique 10-digit identifier called a Community Health Index (CHI) number. This is a further update to our previous publication, where the process of cohort identification has been described [7]. Briefly, all patients with renal cysts diagnosed between January 2009 and December 2019 were recorded in the regional database and reviewed at multidisciplinary meetings. The discussions were recorded on a proforma (Appendix 1), and patients were followed up with interval imaging as necessary. Following consensus at multidisciplinary meetings, category I and II cysts were discharged and the remaining were followed up. In the case of radiological progression, further discussions in multidisciplinary meetings were arranged and treatment plans recorded. For the purposes of investigating complex cysts, Bosniak I and II cysts, haemorrhagic cysts, inflammatory cysts and small renal masses (SRMs) were excluded from analysis. Figure 1 illustrates the study design.

### Data collection

The data from each patient with renal cysts were documented on a secure electronic database and linked to other electronic databases using a unique 10-digit Community Health Index (CHI) number. The databases linked using the CHI number included the National Picture Archiving and Communication System (PACS), Clinical Portal, Integrated Clinical Environment (ICE), Referral Management System (RMS) and Electronic Discharges from hospital systems.

The deterministic records-linkage methodology between systems provided us with the time to follow-up, time to progression/regression, details of surgical intervention and the data of the resected specimen such as histologic subtype, grade and stage.

### Imaging protocol

**Fig. 2:**
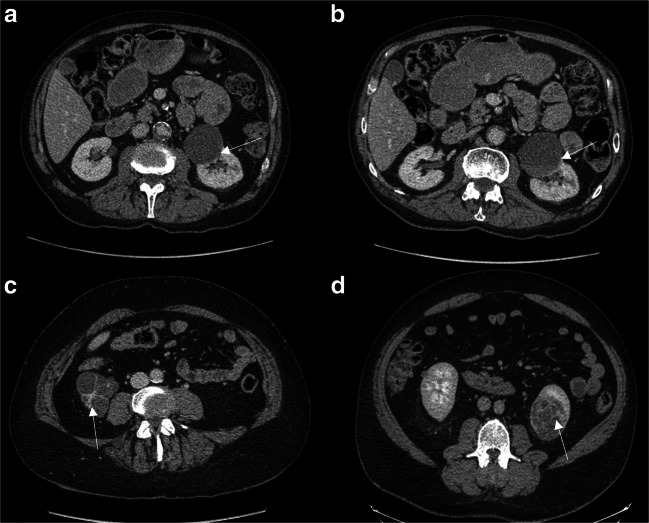
Post-contrast axial CT images of complex left renal cysts. **a** Bosniak IIF cyst progressing to (**b**) Bosniak IV after 6 months with progressing enhancing wall (arrows), clear cell carcinoma, pT1a grade 2. **c** Bosniak III cyst with thick enhancing septae (arrow), clear cell carcinoma, pt1a grade 1. **d** Bosniak IV cyst with thick septa (arrow), clear cell carcinoma, pT1b grade 2

There is an established protocol to characterise cysts at our institution as described before [7]. The CT renal characterisation scan is triphasic with precontrast, arterial (40 s post-contrast) and nephrographic phases (100 s post-contrast) following IV administration of 100 ml of iodine-based contrast agent (Omnipaque) and acquired as a volume using a 64-slice thickness CT scanner (Fig. 2). The 100-s delay helps to visualise the kidneys in their nephrographic phase, the optimal phase to characterise renal cysts. The nephrographic phase was also obtained using MRI and gadolinium contrast. T2 HASTE (6-mm slice thickness, 1.8-mm gap), T1 VIBE pre- and post-contrast (3.5-mm slice thickness, 0.7-mm gap), STIR (8-mm slice thickness, 2.4-mm gap) and diffusion-weighted (4-mm slice thickness, 1.2-mm gap; *b* values 50, 400, 1000 s/mm^2^) images were obtained. The change in cyst enhancement was measured by calculating the difference in pre- and post-contrast enhancement.

### Follow-up protocol

Patients with Bosniak IIF cysts were followed up in accordance with the local protocol with serial imaging at 6, 12, 24, 36 and 48 months, preferably using the same imaging modality. In cases of high likelihood of contrast nephropathy, MRI was used for surveillance. The follow-up period was adjusted subject to a patient’s co-morbidities and suitability for surgical intervention. If the follow-up imaging demonstrated an increased complexity of the cyst, the case was referred to the multidisciplinary meeting and the imaging was reviewed by the uroradiologist. Cyst progression was defined by increased cyst complexity resulting in an upgrade in Bosniak category. Radiological findings attributable to cyst progression included progressing nodule enhancement, septal thickening and wall thickening [2].

Patients with Bosniak III and IV cysts were discussed in clinic and surgical intervention was recommended. Surgical intervention included partial or total nephrectomy, radiofrequency ablation or cryoablation, subject to cyst characteristics and patient-specific factors. Poor surgical candidates or those who opted for conservative management were followed up with serial imaging or were discharged, subject to patient preference.

### Interobserver variability

The interobserver agreement was evaluated by comparing the Bosniak category reported on the initial scan report at the time of detection, with the category reported at the multidisciplinary meeting by the specialist uroradiologist. All cysts discussed at the multidisciplinary meeting (Bosniak ≥II) and reported using the Bosniak classification were included. The linear and quadratic-weighted kappa statistics were calculated to evaluate the rate of agreement.

## Results

There were 452 cysts in 415 patients recorded in the database during the study period. After excluding non-complex cysts (Bosniak II cysts were included within the interobserver rate analysis), there were 317 cysts: 161 Bosniak IIF (50.8%), 79 Bosniak III (24.9%) and 77 Bosniak IV cysts (24.3%). Twenty-nine patients had two cysts, two patients had three cysts and two patients had five cysts. The mean cyst size was 3.9 cm at the time of diagnosis (range, 0.3–15.0 cm).

### Interobserver variability

There were 257 cysts categorised on the initial radiology report and followed up at a multidisciplinary meeting (Table 1). The two readers agreed on the classification in 70% of cases (179/257). Overall, there was a good degree of agreement between the two independent radiologists for all Bosniak cysts ≥ II, as demonstrated by a linear and quadratic-weighted kappa statistic of 0.65 (95% confidence interval, 0.58–0.73) and 0.73 (95% confidence interval, 0.54–0.91), respectively.

**Table 1:** Pre- and post-multidisciplinary meeting Bosniak classifications

Bosniak pre-multidisciplinary meeting	Bosniak post-multidisciplinary meeting				
	2	2F	3	4	Total
1	1 (0.4%)	1 (0.4%)	0	0	2 (0.8%)
2	37 (14.4%)	9 (3.5%)	1 (0.39%)	1 (0.39%)	48 (18.7%)
2F	27 (10.5%)	78 (30.4%)	12 (4.7%)	4 (1.6%)	121(47.1%)
3	3 (1.2%)	10 (3.9%)	38 (14.8%)	5 (2.0%)	56 (21.8%)
4	2 (0.8%)	0	2 (0.8%)	26 (10.1%)	30 (11.7%)
Total	70 (27.2%)	98 (38.1%)	53 (20.6%)	36 (14.0%)	257 (100%)

The rates of disagreement for each Bosniak classification were as follows: 47% for II, 20% for IIF, 28% for III and 28% for IV. Rates of disagreement were significantly higher for Bosniak II cysts than ≥ IIF cysts (*p* = 0.01). Bosniak II and IIF cysts were initially over-categorised on 46% (32/70) and 10% (10/98) of occasions, respectively. These were downgraded on review by a specialist uroradiologist at the multidisciplinary meeting.

### Overview of complex cysts

#### Bosniak IIF cysts

There were 161 cysts categorised as Bosniak IIF (mean size, 4.0 cm; range, 0.3–12.7 cm). Of this group, 151 cysts (89%, 139 patients) were followed up with surveillance imaging over a median follow-up time of 21.2 months (range, 3.0–133.8 months). The remaining 10 cysts (6.2%, 10 patients) were discharged immediately from follow-up imaging because the patients were poor surgical candidates.

**Fig. 3:**
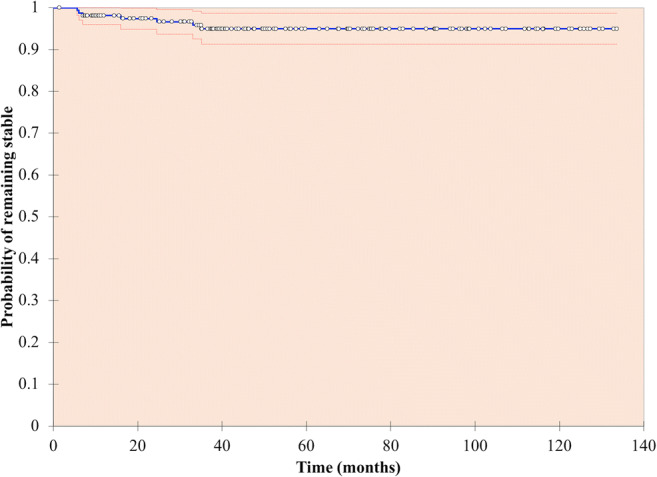
Kaplan-Meier analysis—probability of Bosniak IIF cysts remaining stable

Seven cysts (4.6%) progressed after a median follow-up time of 15.5 months (range, 5.6–35.1 months), 6 of which progressed to Bosniak III (4.0%) and 1 progressed to Bosniak IV (0.7%) (Fig. 3) (Table 2). Five of the 7 progressed cysts were surgically resected, one cyst was treated with cryoablation without a biopsy and one cyst was suitable for surveillance and regressed to a Bosniak IIF after 42.4 months. The malignancy rate of the surgically treated cysts was 60% (3/5), and all malignant cysts progressed within 16 months. These were completely resected, low ISUP grade and stage, and there was no recurrence.

**Table 2:** Progression and regression rates

Initial category	Final category					
	1	2	2F	3	4	Total
2F	4 (2.6%)	1 (0.7%)	139 (92.1%)	6 (4.0%)	1 (0.7%)	151
3	3 (7.7%)	2 (5.1%)	1 (2.6%)	32 (84.8%)	1 (2.6%)	39
4	2 (11.1%)	0	0	1 (5.6%)	15 (83.3%)	18
Total	9	3	140	39	17	208

Five cysts (3.3%) regressed after a median follow-up time of 9.8 months (range, 2.1–18.7 months) (Table 2). Cysts smaller than 2.0 cm were more likely to regress than larger cysts (*p* < 0.01). The remaining 139 cysts (92.1%) remained stable.

#### Bosniak III cysts

There were 79 Bosniak III cysts in 71 patients (mean size, 4.37 cm; range, 1.0–11.5 cm). Twenty-four cysts (30.4%) were resected immediately and five cysts underwent delayed resection after a period of surveillance. One cyst in a transplanted kidney (1.3%) was amenable to cryoablation and was treated without biopsy. The malignancy rate of surgically resected cysts in this category was 79.3% (23/29), and the malignancy rate in the delayed surgery group was 100% (5/5).

Fifty-four cysts (68.4%) were initially managed conservatively, 39 of which (72.2%) were followed up under surveillance. Of the cysts that were followed up, 32 cysts (82.1%) did not progress in complexity or size after median follow-up of 20.5 months. One cyst (2.6%) progressed in complexity to a Bosniak IV category and was deemed suitable for surveillance. Four cysts (10.3%) increased in size after a median follow-up period of 30.1 months and underwent delayed resection. One cyst was visualised as two separate Bosniak III cysts on a follow-up scan, and both were resected after 21.1 months of surveillance. Six cysts regressed (15.4%) with a median time to regression of 15.8 months (range, 6.6–29.0 months).

The most common reasons for conservative management included preference for surveillance in 13 cysts, poor surgical candidates because of co-morbidities in 11 cysts (5 of which were discharged immediately from follow-up imaging), 12 cysts being suitable for surveillance and 10 cysts which were stable from previous imaging (Table 3).

**Table 3:** Indications for conservative management of Bosniak III and IV cysts

Indications for conservative management	Bosniak III	Bosniak IV	Total
SRM suitable for surveillance	12 (15.2%)	11 (14.3%)	23 (14.7%)
Poor surgical candidate	11 (13.9%)	13 (16.9%)	20 (12.8%)
Patient preference	13 (16.5%)	2 (2.6%)	15 (9.6%)
Stable or regressing after reviewing previous imaging	10 (12.7%)	0 (0.0%)	10 (6.4%)
Lost to follow-up	3 (3.4%)	4 (5.2%)	7 (4.5%)
Multiple bilateral cysts	5* (6.3%)	0 (0.0%)	5 (3.2%)

*SRM* small renal mass

*One patient had a total of 5 cysts bilaterally

#### Bosniak IV cysts

Seventy-seven Bosniak IV cysts were identified in 77 patients (mean size, 4.01 cm; range, 1.0–9.9 cm). Forty-three cysts (55.8%) were resected immediately after MDT discussions, three cysts (3.9%) were resected after a period of surveillance and four cysts (5.2%) were treated with radiofrequency ablation without a pre-operative biopsy. The malignancy rate in the surgically resected group was 84.8% (39/46), and the malignancy rate of cysts which underwent delayed resection was 66.7% (2/3).

Thirty cysts (39.0%) were managed conservatively, 18 of which (60%) were followed up. Of the cysts that were followed up, 10 cysts (55.6%) did not progress in complexity or size after a median follow-up time of 25.3 months (range, 11.6–84.2), five cysts (27.8%) increased in size after a median follow-up period of 35.2 months and three cysts (16.7%) regressed after a median follow-up time of 18.6 months (Table 2). Three of the five cysts that were increasing in size underwent delayed resection, one was treated successfully with radiofrequency ablation without biopsy and one remained under surveillance. The most common reasons for conservative management included 11 cysts being suitable for surveillance, 13 patients (13 cysts) being poor surgical candidates and 4 cysts lost to follow-up (Table 3).

### Analysis of the resected cysts

The rate of surgical intervention for IIF, III and IV cysts was 3.1% (5/161), 36.7% (29/79) and 59.7% (46/77), respectively. Of the resected cysts, 6.3% (5 cysts) were progressed IIF cysts, 36.3% (29 cysts) were Bosniak III cysts and 57.5% (46 cysts) were Bosniak IV cysts. In 53% of cases, a partial nephrectomy was performed; in the remaining 47%, a total nephrectomy was performed.

The overall rate of malignancy in surgically resected cysts was 81.3% (65/80) (Table 4). Of the malignant tumours, histopathology confirmed that 73.8% were clear cell, 13.8% were papillary, 6.2% were multilocular cystic carcinomas and the remaining were either chromophobe or collecting duct carcinomas. The malignancy rate in cysts that were resected after a delay versus those resected immediately was 76.9% (10/13) and 82.1% (55/67), respectively, and this was not statistically significant. Of all malignant tumours, 73.8% were of low ISUP grade, 93.7% were confined to the kidney (stage pT2b or less) and 81.0% were less than 7 cm (stage pT1a or pT1b). The recurrence rate was 1.5% (1/65) after a median follow-up time of 50.2 months (range, 2.8–93.1 months). The grade and stage of the tumours were not statistically different between those who had immediate or delayed surgery.

**Table 4:** Histology of surgically resected cysts

Outcome of operation	Bosniak IIF (*N* = 5)	Bosniak III (*N* = 29)	Bosniak IV (*N* = 46)	Total (*N* = 80)
Clear cell RCC	2 (40%)	14 (48.3%)	32 (69.6%)	48 (60%)
Papillary RCC	0	4 (13.8%)	5 (10.9%)	9 (11.3%)
Multilocular cystic RCC	1 (20%)	3 (10.3%)	0	4 (5.0%)
Chromophobe RCC	0	2 (6.9%)	1 (2.2%)	3 (3.4%)
Collecting duct carcinoma	0	0	1 (2.2%)	1 (1.3%)
Multilocular cystic nephroma	0	1 (3.4%)	0	1 (1.3%)
Oncocytoma	0	0	3 (6.5%)	3 (3.4%)
Mixed epithelial and stromal	0	0	1 (2.2%)	1 (1.3%)
Benign cyst	2 (40%)	5 (17.2%)	3 (6.5%)	10 (12.5%)

## Discussion

### Key findings

Overall, there is a good degree of agreement between radiologists using the Bosniak classification system; however, Bosniak II cysts are frequently over-graded. Both the rate of progression and regression of Bosniak IIF cysts are low (4.6% and 3.3%, respectively), and malignant cysts progress during the early surveillance period (within 16 months). Surgically resected Bosniak III and IV cysts have a high rate of malignancy, but the rate of benign cyst resection is still high (18.8%). Malignant cysts on histopathology showed low-grade and early-stage renal cancers.

### Study findings in relation to relevant literature

The consensus from the literature is that a surveillance period is safe and facilitates identification of Bosniak IIF lesions that require surgical intervention [9]. A definitive surveillance period for Bosniak IIF cysts has not been settled upon within the literature, but a period of up to 4 years has been suggested [1, 6, 9]. Our data suggests that malignant progression and regression in most cases occur early in the surveillance period and a shorter surveillance period would be acceptable. Of course, the possibility of later malignant progression remains, albeit very small and was not observed in this population.

There is no universally accepted interval period between the surveillance scans for Bosniak IIF cysts [16]. In our study, the Bosniak IIF cysts were followed up in accordance with the local protocol with serial imaging at 6, 12, 24, 36 and 48 months, subject to patient factors. A total of 287 follow-up scans were performed on these cysts, and seven instances of progression were reported. Although the malignancy rate of progressed IIF cysts is high, all three of the malignant Bosniak IIF cysts were early stage and low grade; thus, a greater interval between scans may be appropriate [17]. One study suggests that a longer interval period permits cysts sufficient time to develop radiological features and reduces radiation exposure [18]. The rate of progression of Bosniak IIF cysts within the literature ranges from 4.6 to 15.6% [5–7]. In the present study, the progression rate was low (4.6%) and supports a shorter follow-up period and a greater interval time between surveillance scans.

The unnecessary resection of benign Bosniak III cysts is a recognised limitation of the Bosniak classification. Prior to the introduction of the IIF category, the malignancy rate of Bosniak III cysts had been reported as low as 31% and 45% [3, 11]. The addition of the intermediate IIF category has improved the clinical significance of the Bosniak classification, as evidenced by increased malignancy rates of Bosniak III cysts and decreased resection rates. Studies have since reported malignancy rates between 60 and 81.8% [8, 19]. Graumann et al conducted a meta-analysis of 15 retrospective studies and found that the malignancy rate was 65.4% [20]. In the present study, we report a malignancy rate of 79.3% and six resected Bosniak III cysts (20.7%) were benign. The revised Bosniak classification published by Silverman (2019) et al aimed at reducing the frequency of benign cyst resections by incorporating explicit definitions of terms and specific inclusion criteria. It defines the number of septae, thickness of wall and septae and nodularity for each category and sets the criteria to increase the specificity of the classification system [21]. These features were not tested in the present study, and whether the revised Bosniak classification of 2019 improves the specificity of categorisation in predicting malignancy remains to be seen. We envisage that adoption of more qualitative and quantitative criteria with machine learning algorithms would certainly reduce the frequency of benign cyst resections, improve our ability to follow up these lesions on active surveillance and increase the malignancy rate of resected cystic renal masses. Future research is required to externally validate the 2019 classification.

The ISUP grading system is employed to predict the biological aggressiveness of the cancer, and an ISUP grade of 1 or 2 predicts a relatively indolent cancer. Of the resected Bosniak III and IV cysts, 89.5% and 69.2% were of low ISUP grade, respectively. These findings correlate with a low grade of renal cell carcinomas in the literature irrespective of Bosniak category [13, 14]. Given the relatively indolent nature of the malignant cysts, survival analysis studies with long-term follow-up would be useful to identify patient sub-groups who would benefit from surgical intervention or surveillance management, particularly in the Bosniak III and IV groups. Surgical decision-making in the management of complex renal cystic disease should also consider patient-related factors such as age and number of co-morbidities in addition to the risk of malignancy based on classifications of structural abnormalities on imaging. Any radiological classification can only predict risk of malignancy and not biological behaviour of renal cancers.

In our study, there was no correlation between delayed surgery and higher grade. All cysts that underwent delayed surgery were of low grade, and the period of observation did not compromise outcome. A period of observation may help in selecting progressing cysts, increase the malignancy rate of resected Bosniak III cysts and reduce the proportion of benign cyst resections. Further studies are warranted to investigate if a period of observation would reduce the rate of benign surgery and not compromise cancer outcomes.

## Conclusion

This study further confirms that a good degree of agreement exists between radiologists in classifying complex renal masses using the Bosniak classification. The progression rate of Bosniak IIF cysts is low, but the malignancy rates of progressed Bosniak IIF cysts and Bosniak III and IV cysts are high. A very high proportion of histopathologically confirmed cancers in complex renal cysts are of low grade and stage.

## Electronic supplementary material

ESM 1 (DOCX 477 kb)

## Abbreviation

ISUP: International Society of Urological Pathology
