# Supplementary figures and images for: Complex renal cysts (Bosniak ≥IIF): interobserver agreement, progression and malignancy rates
Source: Eur Radiol. 2020 Aug 27;31(2):901–8. doi: 10.1007/s00330-020-07186-w (PMC7813744; doi:10.1007/s00330-020-07186-w)

**Appendix 1**

*Appendix 1. Multidisciplinary meeting proforma.*


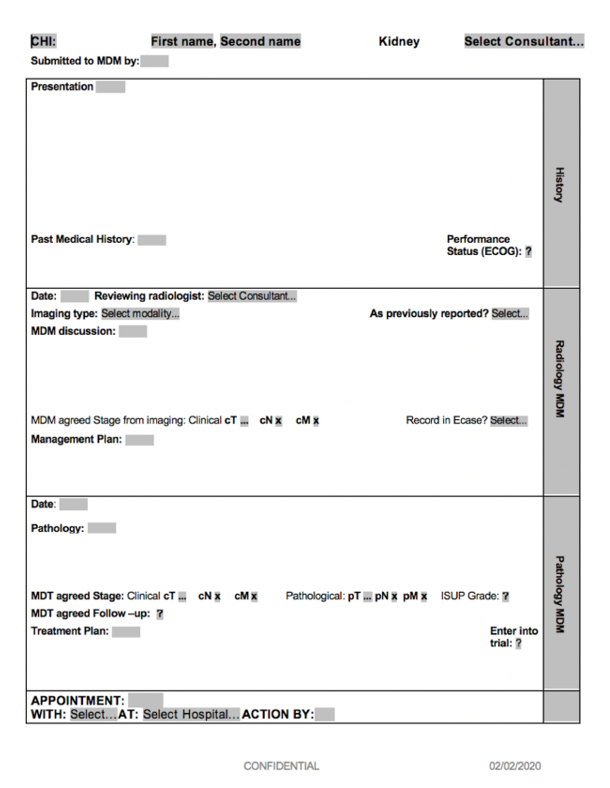

Supplement: Supplementary file 1 — (DOCX 477 kb) [file 330_2020_7186_MOESM1_ESM.docx]
